# Zurbarán attribution hypothesis supported by pigment analysis and multiband images observation of four paintings by his workshop

**DOI:** 10.1038/s41598-023-27677-2

**Published:** 2023-01-16

**Authors:** Javier Moreno-Soto, Anabelle Križnar, Francisco José Ager, María Auxiliadora Gómez-Morón, Antonio Gamero-Osuna, Agustín Martín-de-Soto, Miguel Ángel Respaldiza

**Affiliations:** 1 Departamento de Física Aplicada I, Escuela Politécnica Superior, Universidad de Sevilla, C/ Virgen de África 7, 41011 Sevilla, Spain; 2 Centro Nacional de Aceleradores, Universidad de Sevilla-Junta de Andalucía-CSIC, Avenida Tomas Alva Edison 7, 41092 Sevilla, Spain; 3 Departamento de Escultura e Historia de las Artes Plásticas, Facultad de Bellas Artes, Universidad de Sevilla, C/ Laraña 3, 41003 Sevilla, Spain; 4 Instituto Andaluz del Patrimonio Histórico, Camino de los Descubrimientos s/n, 41092 Sevilla, Spain; 5 Taller de restauración del palacio arzobispal, Delegación diocesana de Patrimonio Cultural, Archidiócesis de Sevilla, Plaza Virgen de los Reyes s/n, 41004 Sevilla, Spain; 6 Departamento de Física Atómica, Molecular y Nuclear, Universidad de Sevilla, Av. De Reina Mercedes s/n, 41012 Sevilla, Spain

**Keywords:** Physics, Applied physics

## Abstract

Francisco de Zurbarán was one of the greatest painters of the Spanish Golden Age, with artworks scattered all over the world. Unfortunately, there are hardly any exhaustive studies on the pigments that he used. In this work, four canvas paintings attributed to the Zurbarán Workshop were studied. Each of them presents the figure of a different saint in a particular isolation scene: *Saint Bruno*, *Saint Francis of Assisi*, *Saint Dominic of Guzman,* and *Saint Peter Martyr*. Nevertheless, the painting of *Saint Peter Martyr* shows superior quality in both technique and colours, so it is not clear whether this canvas was also made by the Workshop or by Zurbarán himself, as some art historians claim. Coinciding with conservation and restoration works, the paintings were initially analysed with non-invasive techniques such as ultraviolet photography (UV), infrared reflectography (IRR) and X-ray fluorescence (XRF) to determine the characterization of the pigments. Those studies were complemented by stratigraphic analysis of some extracted samples with optical microscopy (OM) and scanning electron microscopy with energy dispersive X-ray spectroscopy (SEM–EDX). Among a number of results obtained, we found significant differences between the pigment palette used in *Saint Peter Martyr* and the other paintings, supporting the hypothesis that this painting was done by Zurbarán himself instead of his workshop. These results could also help to distinguish other authentic paintings by the master’s hand from very similar paintings by his workshop or by other artists.

## Introduction

Francisco de Zurbarán (1598–1664) was one of the great painters of the Spanish Golden Age^1^. The artist excelled in religious painting during a period of expansion of religious communities and the flourishing of convents. He always remained within the Tenebrist current of the early seventeenth century and his work is characterized by light effects influenced by Caravaggio^2^. Throughout his work, it was typical for Zurbarán to paint individual figures with a particular isolation scene without spatial reference. In addition, many similarities in his work can be observed, especially in his series of saints. This was due to the commissions that he received from different religious orders, first in Spain and later in America, to spread their images of saints as founders of Monastic Orders in the New Territories.

**Figure 1:**
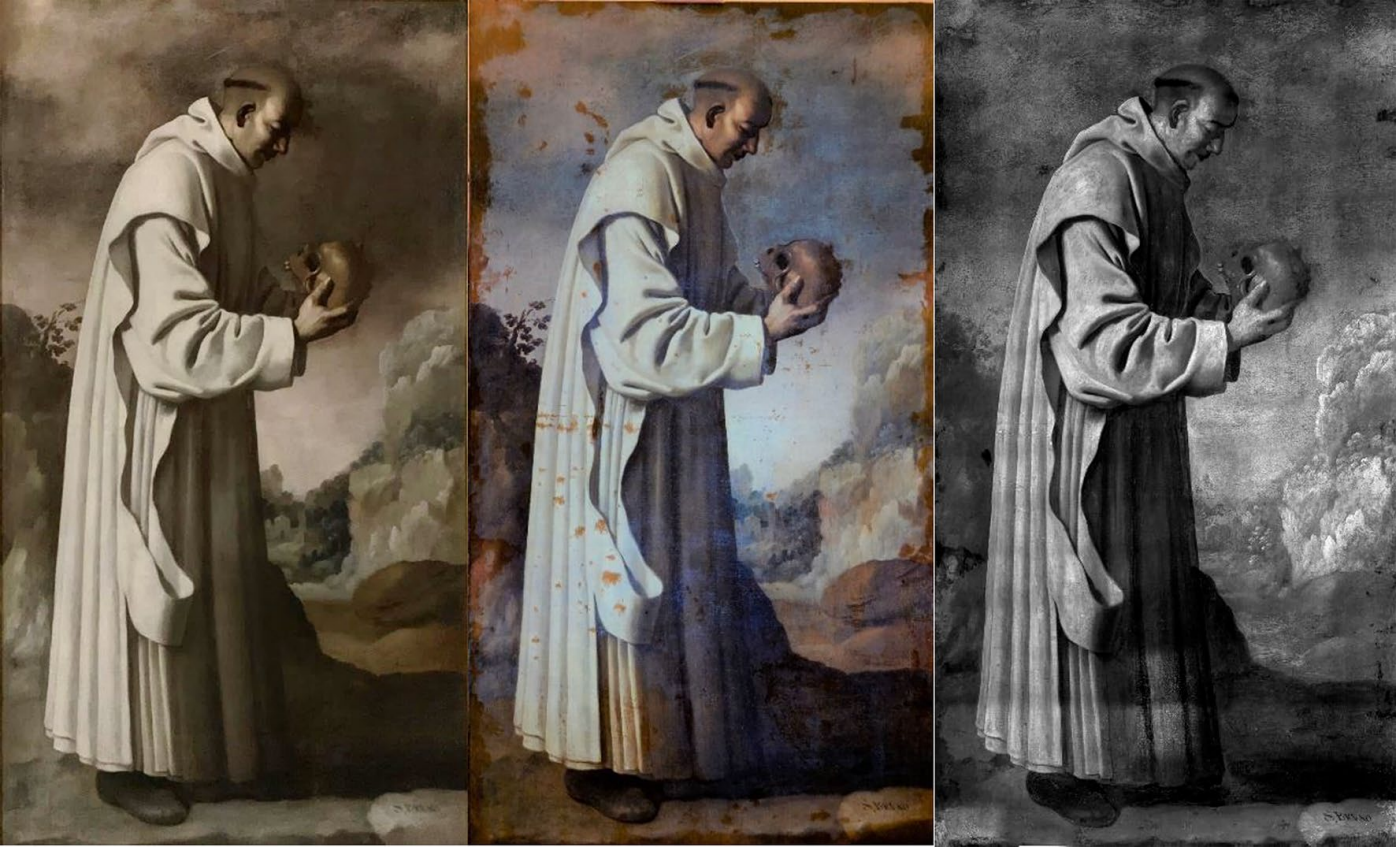
Painting of *Saint Bruno* (*SB*) in visible (left), UV (centre), and IR (right) light.

**Figure 2:**
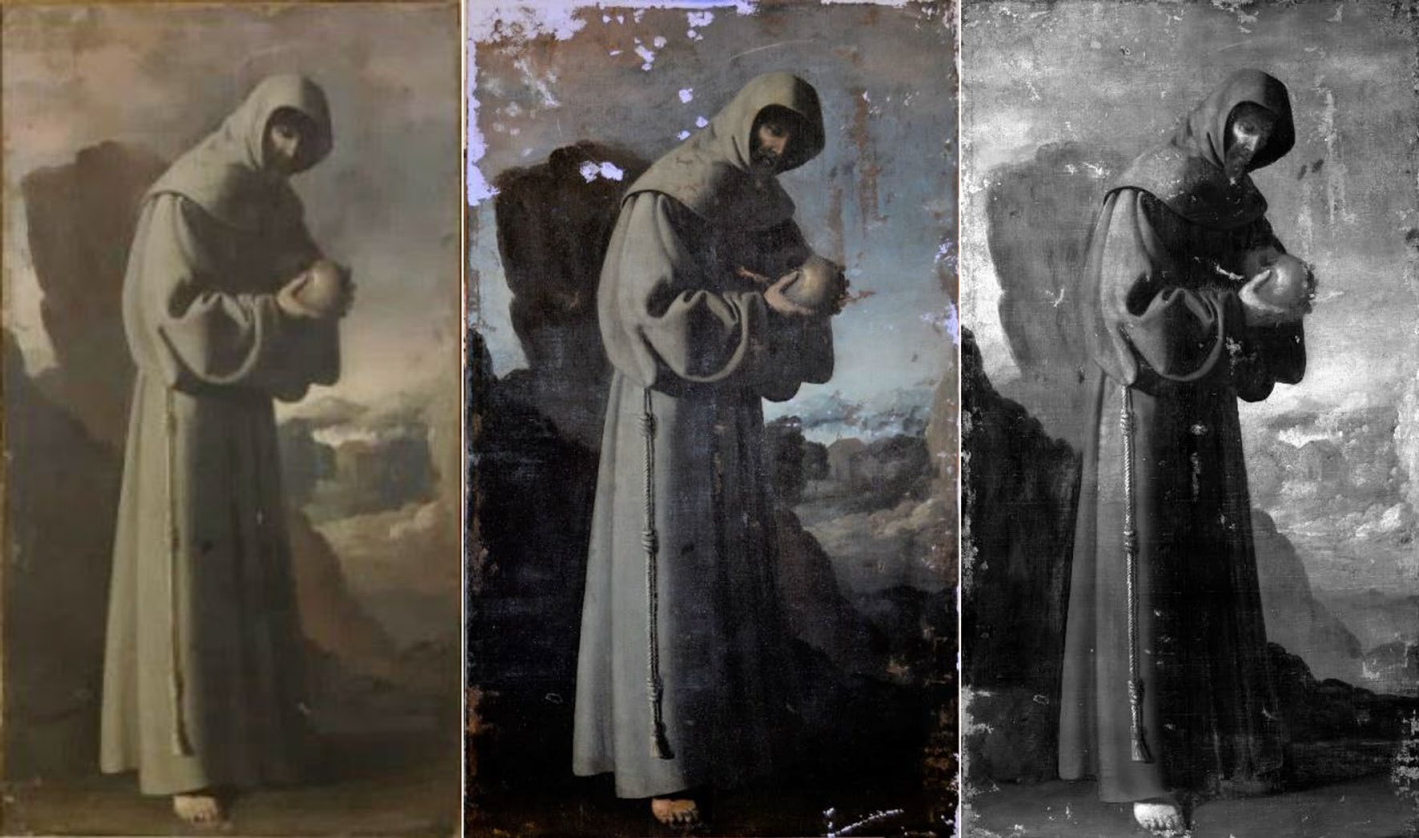
Painting of *Saint Francis of Assisi* (*SF*) in visible (left), UV (centre), and IR (right) light.

**Figure 3:**
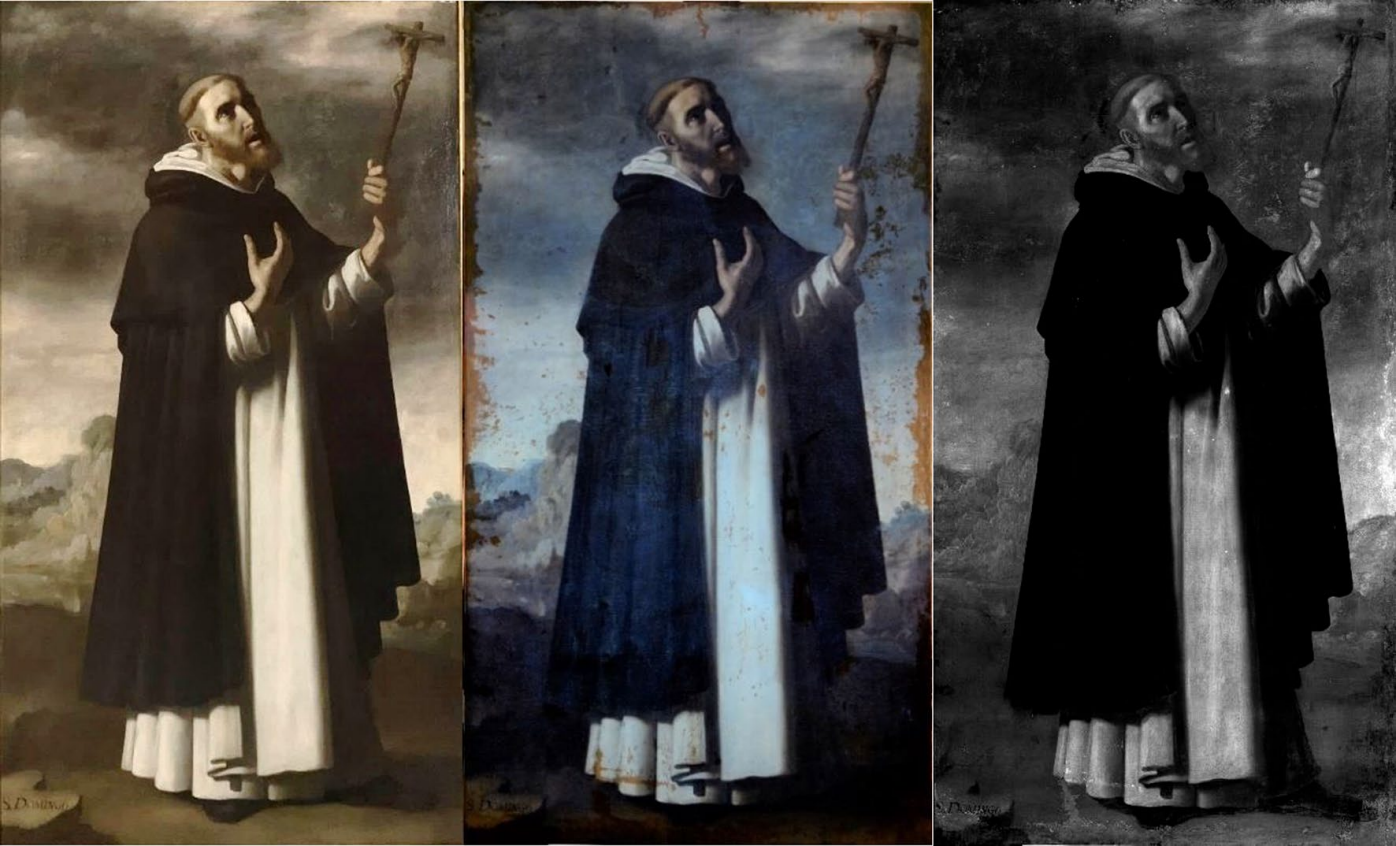
Painting of *Saint Dominic of Guzman* (*SD*) in visible (left), UV (centre), and IR (right) light.

**Figure 4:**
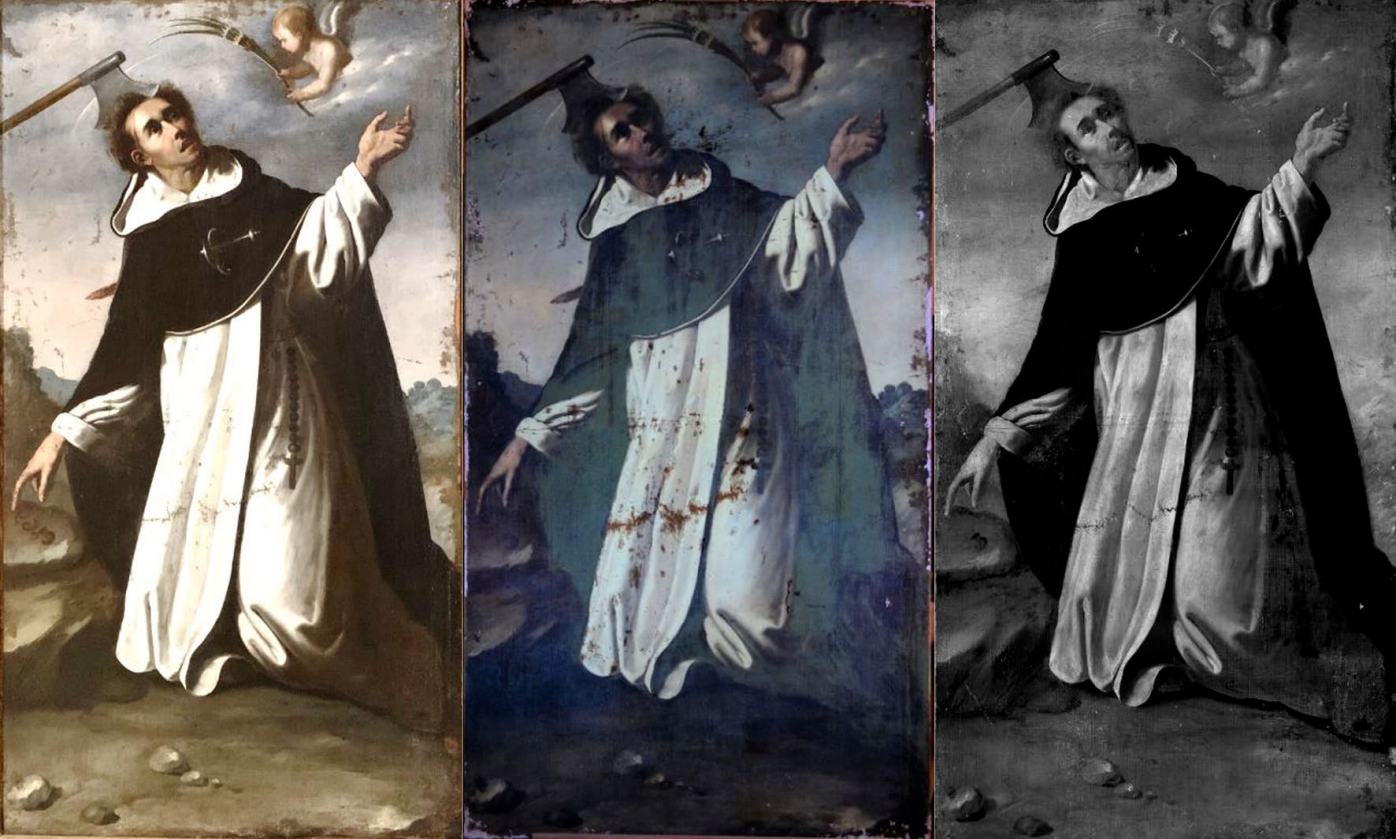
Painting of *Saint Peter Martyr* (*SP*) in visible (left), UV (centre), and IR (right) light.

The collection of the Archbishop’s Palace of Seville conserves six artworks attributed to Zurbarán or his workshop, of which four were studied in this work: *Saint Bruno* (*SB*), *Saint Francis of Assisi* (*SF*), *Saint Dominic of Guzman* (*SD*), and *Saint Peter Martyr* (*SP*), see Figs. 1, 2, 3 and 4 (left). This work was part of the Archbishop’s Palace project dedicated to the maintenance and conservation of its heritage. The history and origin of the acquisition of these paintings by the Archbishop’s Palace are currently being investigated by their art historians; until now, it is only known that they entered the collection in the nineteenth century, having been mentioned in several inventories. Several experts on this Baroque artist attributed these canvas paintings to his workshop, but nothing has been published so far. Recently, the art historians of the Archbishop’s Palace have supported the hypothesis that SP was painted by Zurbarán, while the other three canvases are a production of his workshop. This painting shows a more refined technique with substantial changes in the composition with respect to the other three canvases, and it presents greater movement and expressiveness, as well as a higher visual intensity in the colour.

The four paintings reflected a great variety in finishes and general states of conservation. Different analytical techniques were used during the conservation-restoration process. The aim of this study was to discover different interventions and a possible preparatory drawing from multiband images, and to obtain detailed knowledge of the pigments used in different paint layers, in order to provide support to the restoration process. In addition, a comparison of these four paintings was planned to confirm or reject the authorship hypothesis of the *SP* canvas.

## Materials and methods

All four paintings were analysed mainly with non-invasive techniques. We used the portable equipment developed at the *Centro Nacional de Aceleradores* (CNA), which allowed us to analyse the paintings in situ in the Archbishop’s Palace.

Preliminary observations were made with two well-known methods: ultraviolet photography (UV) and infrared reflectography (IRR). The first method is based on the different fluorescence produced by the aged and new materials after exposure to UV light. The UV image offers information about interventions in the original painting, and it identifies and locates over-paintings and new varnishes, among others^3–7^. The second method, the IRR technique, is based on the differences in the absorption of IR light by pigments. IR light penetrates deeper into the colour layers, making it possible to visualize some underdrawings and paint changes (*pentimenti*)^5–10^. For UV images, four Wood lamps were placed at both sides of the paintings at a suitable distance (approximately 1 m) to homogeneously illuminate the painting surface. The photography was taken with a Nikon D3X camera, at 400 ISO and 20 s of exposure time. For the IRR images, the painting was illuminated with two 800 W Halogen SDI lamps whose power and position were adjusted to obtain the best results. The camera used was a Xenics Xeva-XS 512 with InGaAs detector that was mounted on a 2D robotic platform by Optimind at 32 cm from the painting to obtain high precision in the camera movement. Movement in the vertical and horizontal directions was controlled by the computer, and the images were captured automatically. Approximately 119 pictures were taken for each painting, and complete IRR images were made with the ImageJ program.

The material study was performed with X-ray Fluorescence (XRF). The XRF technique identifies the chemical elements of pigments and other inorganic materials applied in artworks by the energies of their characteristic X-ray peaks^6,11–14^. Thus, it is possible to obtain a general idea of the palette used by the artist without touching the paintings. The portable XRF equipment consisted of an X-ray generator RX38 from EIS S.L. with a W anode and a Silicon Drift Detector (SDD) with an energy resolution of 140 eV at Mn-Kα line. A 1 mm Al filter was placed on the X-ray generator window to suppress the characteristic W peaks of the anode. Both devices were fixed to a metal structure that allowed forward and backward movement to get closer to the painting with precision and reduce the risk of accidents. In addition, the intersection of two lasers coupled to the system was used to keep the measuring distance fixed. Each measurement was carried out under the same conditions to be able to directly compare the different spectra: the generator was operated at 33.4 kV and 80 µA, and the acquisition time was 200 s. Different colours and tonalities were semi-quantitatively analysed using the areas of XRF peaks. These areas are roughly proportional to the weight concentration of the element. Therefore, a comparison of the content of one element in different areas of similar composition can indicate higher or lower presence of a certain pigment. Nevertheless, the density/weight of the elements must also be taken into consideration, reflected as higher or lower peaks depending on a lighter or heavier chemical element. A total of 119 areas covering the entire surface of the canvases were analysed.

A few small samples were extracted from doubtful areas because of the limitations of these non-invasive techniques. The XRF system used gives information only on chemical elements with an atomic number greater than 14 at the irradiated point; therefore, it is impossible to identify organic pigments or pigments characterized by the same chemical element because there is no molecular information. A total of 28 samples of different areas of the paintings were prepared as cross-sections and analysed by optical microscopy (OM) and scanning electron microscopy with energy dispersive X-ray spectroscopy (SEM–EDX). These techniques give information on the sequence and thickness of the colour layers, their composition, and the granulometry of the pigment^14–16^. OM analysis was performed with a LeicaDM4000M optical microscope, which has a UV light to differentiate interventions and some pigments, while SEM–EDX was performed with SEM equipment developed by JEOL 5600 LV and EDX by Oxford, Inca X-sight, after coating the samples with a thin gold layer for improved imaging.

## Results

### UV and IRR examination

First, the four paintings were examined with UV and IRR techniques. The UV image of *SF* was taken during the stuccoing process of the restoration. The UV images of the paintings, shown in Figs. 1, 2, 3 and 4 (centre), revealed many interventions, made at different times and with different materials, indicated by various tonalities of UV fluorescence. Most interventions were detected on the edges of the paintings, since these areas are usually the most damaged, and on the vestments of *SP*, *SB*, and *SD*. There is no recorded information on any intervention, but the criteria taken in the restoration process presume that many retouches were made after the second half of the twentieth century^17^. The canvases present the same relining but show different states in the degree of intervention. Currently, the four paintings are being restored; restorations and varnish are being removed, while the old, coloured *stucco* and the relining is kept. The final result aims to unify their esthetical vision and state of conservation.

The sky of *SB*, *SF* and *SD* presented a bluish shine in the UV in comparison with the dark tonality of the visible spectrum. The material study shown in the following sections identified the pigment used for the sky as smalt. This blue pigment turns dark for various reasons^18–21^. However, the UV images seem to recover the blue tonality, offering a possible vision of the original sky colour.

**Figure 5:**
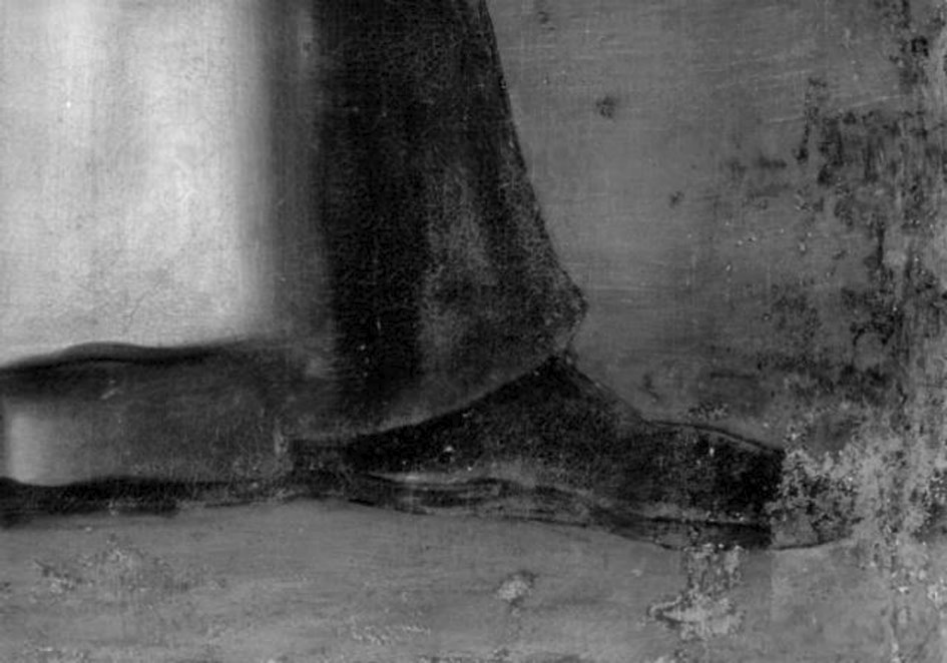
Detail of the IRR image of the left shoe of *Saint Dominic of Guzman.*

The IRR images in Figs. 1, 2, 3 and 4 (right) also identified some of the intervention areas. However, no preparatory drawings were observed except for the left shoe of *SD*, as seen in Fig. 5. This drawing was probably made during an intervention in which the restorer needed to sketch the shoe due to its loss or excessive cleaning.

### Material study

After the retouches were located, XRF was used to characterise the pigments applied in the elaboration of the different colours and tonalities. In addition, OM and SEM–EDX of the samples were used to complete the information in areas where the XRF results were not conclusive. We studied both the original pigments and those used in the interventions. In addition, a comparison between the paintings was carried out to find differences that could indicate different authors.

#### Preparation and priming

All areas analysed with XRF showed the presence of Ca, Fe, and Pb peaks, which may indicate that these elements belong to the preparation and primer layers. The intensity of the peaks depends on the thickness of the superimposed pictorial layers and the pigments used. A Ca-based preparation was confirmed by the analysis of the areas with polychromy loss, in which the preparation or the canvas could be seen with the naked eye. Calcium indicates the application of calcium carbonate (chalk) or calcium sulfate (gypsum) as materials for preparations, as gypsum was generally used in Spanish Golden Age painting^22,23^.

**Figure 6:**
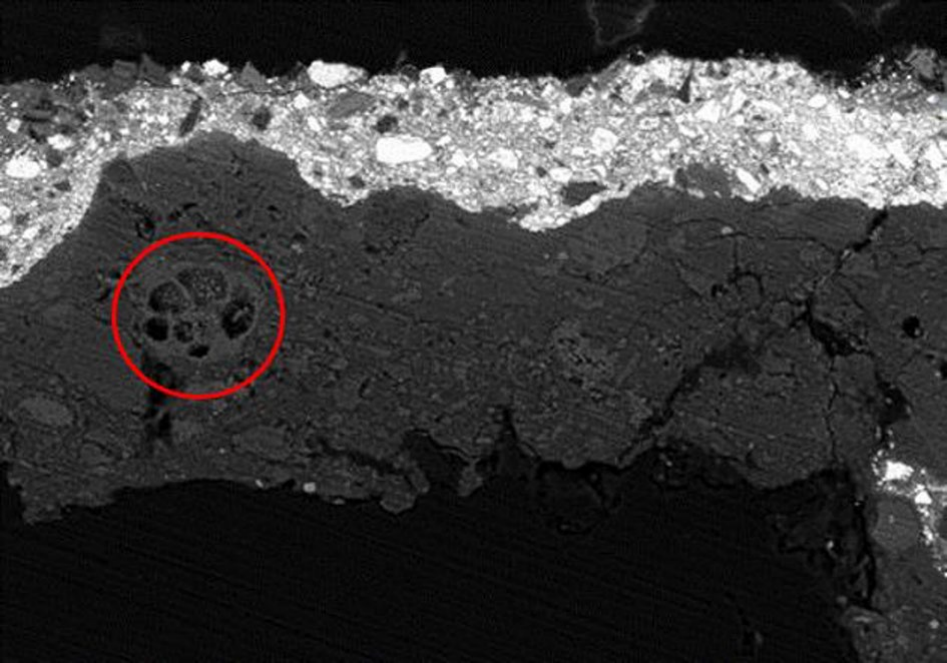
Scanning electron microscope photography in backscattered electron mode. A fossilized shell of calcium carbonate can be observed (red circle).

OM and SEM–EDX analysis of the extracted samples clarified the composition of the preparation, made with gypsum and calcite (Ca), mixed with earths (Fe) and a small amount of lead white (Pb). In the painting of *SD*, a fossilized shell was discovered in the preparation layer, see Fig. 6, indicating the use of chalk. However, slight differences were observed in the preparation layers of all four paintings: in *SP*, a few grains of azurite were found in some samples, while in *SF* a few grains of bone black were observed in the areas of darker tonality, especially in the lower part of the painting. We also observed a thin preparation under the *SP* image, while under the *SF* it was quite thick.

On the other hand, the existence of a primer layer could not be confirmed with XRF, although the presence of Pb would indicate a primer made with a lead-based pigment. The cross-sections did not reveal a uniform primer; only in some samples, a lead white layer was observed, which would suggest that the primer was applied locally in specific areas.

#### Original pigments

The four paintings are characterized by a generally cold palette: white, black, green, blue and brown. There are no intense vivid colours except in the *SP* painting, where the palm leaf in the hands of the angel is decorated with intense red and yellow adornments. The chemical elements obtained by XRF analysis of the original pigments in selected areas are potassium, calcium, manganese, iron, cobalt, nickel, copper, arsenic, tin, mercury and lead, whose individual presence and peak intensity depend on the pigment applied.

These elements showed that the pigments used correspond to the contemporary palette^22,24,25^. A lead-based (Pb) pigment identified as lead white was used for white colour. For yellow pigments, the most applied one was yellow earth characterized by Fe, while sometimes a small amount of lead–tin yellow (Pb and Sn) was added to other pigments to obtain desired tonalities. Only in the yellow palm adornment on *SP*, lead–tin yellow was applied as the principal pigment. Generally, the bright, yellow-coloured tonality was not used in these paintings. Similarly, reds were obtained by mixing different pigments, such as vermilion, recognized by Hg peaks, and red earth, in variable proportions. The latter is impossible to differentiate with XRF from yellow earth since both are identified by the same chemical element (Fe), although we can guess the pigment applied from the tonality of the colour. However, the SEM–EDX analysis perfectly recognised the red grains of the red earth in some areas. This technique also allowed the identification of two other red pigments, a red lake and a minium red (Pb), which was only found in the *SP* painting. The red lake can be identified by intense Ca peaks, when applied on the CaCO_3_ substrate, and is easily recognizable with SEM–EDX. In the *SB* painting, cross-sections did not reveal its presence, but it might have been removed during the cleaning processes of earlier interventions, because the lake was generally used for the final glazes. For blue, the main pigment was smalt, determined principally by the presence of Co in combination with other chemical elements (K, Ni, As, and sometimes Bi) depending on the manufacturing process. In addition, the cross sections confirmed the existence of azurite based on the presence of Cu and its characteristic angular granulation of the pigment’s particles. The brown pigment was umber, characterised by the presence of Mn and Fe, and possibly a burnt earth, while the black pigment was confirmed by SEM–EDX as bone black with intense Ca peaks in the dark areas. Finally, XRF analysis identified very strong Cu peaks in all green areas. This element is characteristic of many green pigments used in the seventeenth century (Malachite, Verdigris, among others). However, the cross-sections revealed that in none of the four paintings a green pigment was used. The green colour was achieved with a mixture of azurite, yellow earth, and lead white, sometimes with quartz, as it was quite common in the Spanish painting of the Golden Age and it was also found in the works by Murillo or Pacheco^22,26^. The different pigments used in the different areas of the paintings are explained below.

### “Skin tones and hair”

**Figure 7:**
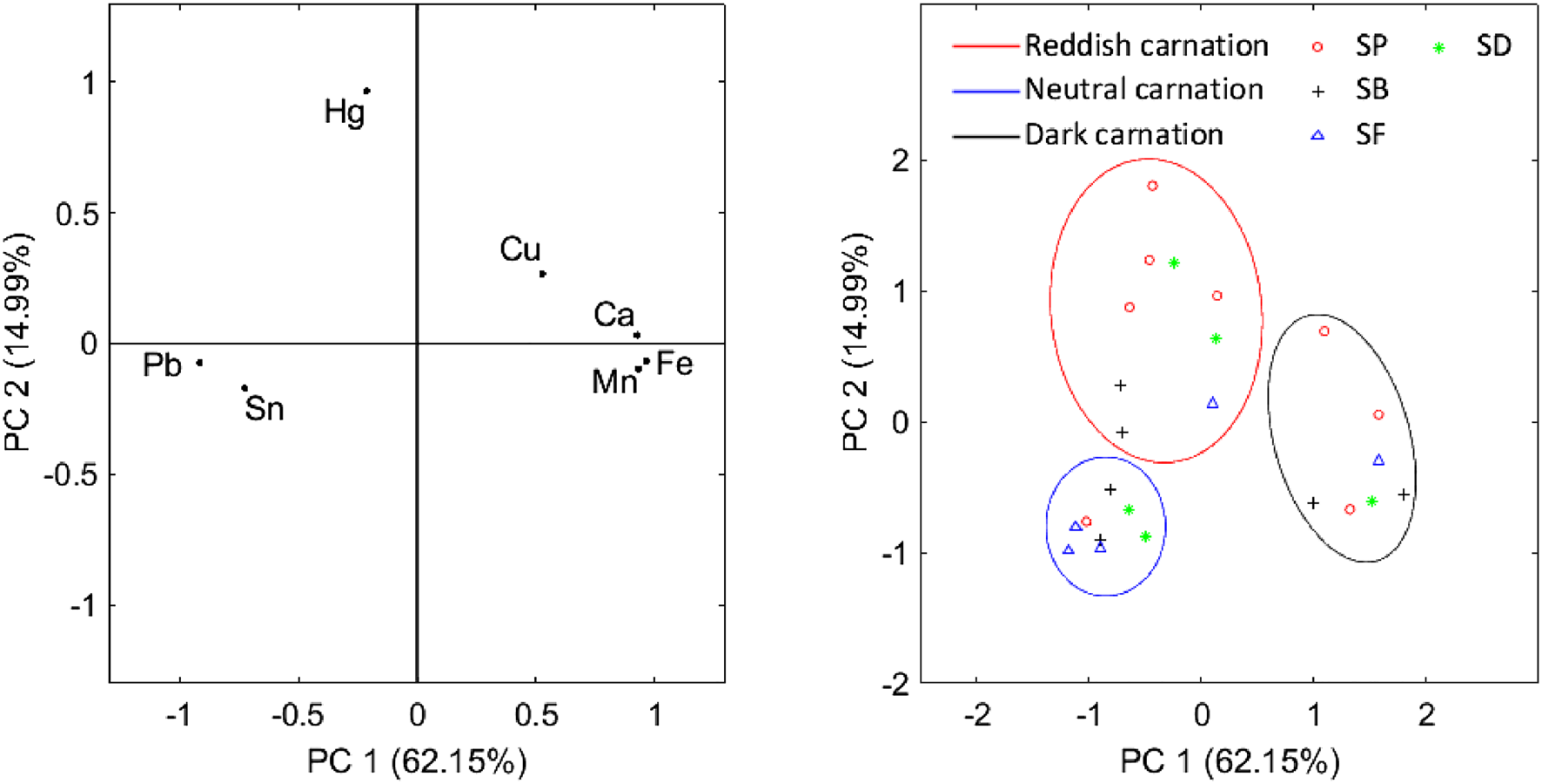
PCA scores (left) and results of the PCA analysis applied to the pigments of the skin tones of the four paintings (right). Three groups are identified based on the tonality of the pigments: neutral skin tone (blue), dark skin tone (black), and reddish skin tone (red).

The basic colour of all skin tones was made with high amounts of lead white mixed with yellow or/and red earth, a very low amount of lead–tin yellow, and a copper-based pigment. As mentioned above, no green pigment was used for the green areas, so azurite could also be applied for skin tones. However, blue pigment was normally used for dead skin colour, while green pigment was generally added to the living skin tone. It was not possible to extract samples from the complexion to better define the Cu-based pigment; therefore, we cannot know if the skin tone contains blue or green copper pigments. The combination of all these pigments and the relative amounts vary depending on the tonality. The highlights showed more lead white and lead–tin yellow, while the shades were made with more earths and a copper-based pigment, together with the addition of umber and sometimes bone black, identified by the presence of large calcium peaks in darker areas. For the reddish tonalities of the cheeks, ears, hands, and lips, vermilion was used. We found that the amount of this red pigment used in the elaboration of the skin tones was lower in the paintings of *SB* and *SF*, whereas in *SD* and *SP* there was much more vermilion (Hg), resulting in a more intense reddish tone. Figure 7 shows the principal component analysis (PCA) applied to skin tone pigments and summarizes everything mentioned above. Three distinct groups can be observed, which correspond to (a) neutral skin tone, (b) dark skin tone that is strongly correlated with bone black (Ca), umber (Mn and Fe) and earths (Fe), and (c) reddish skin tone characterized by a higher presence of vermilion (Hg). In addition, in the reddish tonality, the lowest points correspond to *SB* and *SF*, indicating that a lower amount of vermilion was used.

The hair of the saints and the angel have different tonalities. For the angel, the analysis showed a high presence of yellow earth and umber mixed with vermilion and lead–tin yellow to obtain a brownish blonde colour. For the saints, who have a darker hair, the amount of Pb–Sn yellow decreased, most likely belonging only to the layer of skin tone underneath, while vermilion was not found for *SB*. Furthermore, all the spectra of dark hair showed intense Ca and Cu peaks, much higher than in other areas of the painting, indicating that a bone black or red lake and a copper-based pigment were used, especially for the shades.

### “Vestments and objects”

The vestments of the saints are composed of a white tunic, except for *SF*, which has a greyish colour, and a black cape in case of *SD* and *SP*. The white tunics were painted with lead white, while the grey tunic of *SF* was darkened with ochres and umber. Different amounts of these last two pigments were used to create the shades in the four tunics. However, analysis by SEM–EDX showed that different pigments were added for each tunic. For *SB* and *SF*, the dark areas were made by adding a small amount of vermilion and bone black, respectively, while in *SP* azurite was detected and in *SD* only earths were observed. However, the cincture of the *SF* habit was painted with lead–tin yellow with a decrease in dark pigments. The black capes of the two saints were essentially made up of bone black, earths, and umber with a small amount of azurite.

The objects in the hands of the saints have a brownish colour, which was obtained mainly with an earth mixed with a small quantity of umber, a copper-based pigment, and a variable quantity of lead white. Furthermore, a small presence of vermilion was found in the skull of *SF*, probably from a lower layer. For the lighter and darker areas, lead–tin yellow and bone black were added, respectively. In the *SP* painting, the rosary is composed of the same pigments as the skull in *SF* but with more vermilion, while the grey colour of the sword and axe was painted with lead white, earths, smalt, and azurite. Finally, for the palm tree, a green colour was used that, as mentioned previously, was achieved with a combination of azurite, yellow earth, lead white, and red lake. The red and yellow ornaments that showed the most intense colours of all the paintings were made with vermilion and lead–tin yellow.

### “Sky”

The sky was painted with a mixture of lead white and smalt, whose quantity varies depending on the tonality of the sky. In addition, Fe was detected in all analysed points. Therefore, an earth was probably used in the underlying layer to give more body to this vitreous pigment, or it was added directly to the smalt for darker areas. The smalt is a very unstable pigment that loses its blue colour and turns brown, probably due to its reaction with the binder or varnish^18–21^. In the *SP* painting, a large amount of Cu was found, indicating the use of azurite together with smalt. This is probably the reason that this background presents a more intense blue sky than the other three. Red pigments were also included in some reddish areas, but the use of these pigments varied between the paintings, as shown by SEM–EDX: in *SF*, vermilion and red lake were used; in *SP*, vermilion and minium red; in *SB* only vermilion; and, in *SD,* only red lake^18–21^.

### “Vegetation, rocks, and ground”

**Figure 8:**
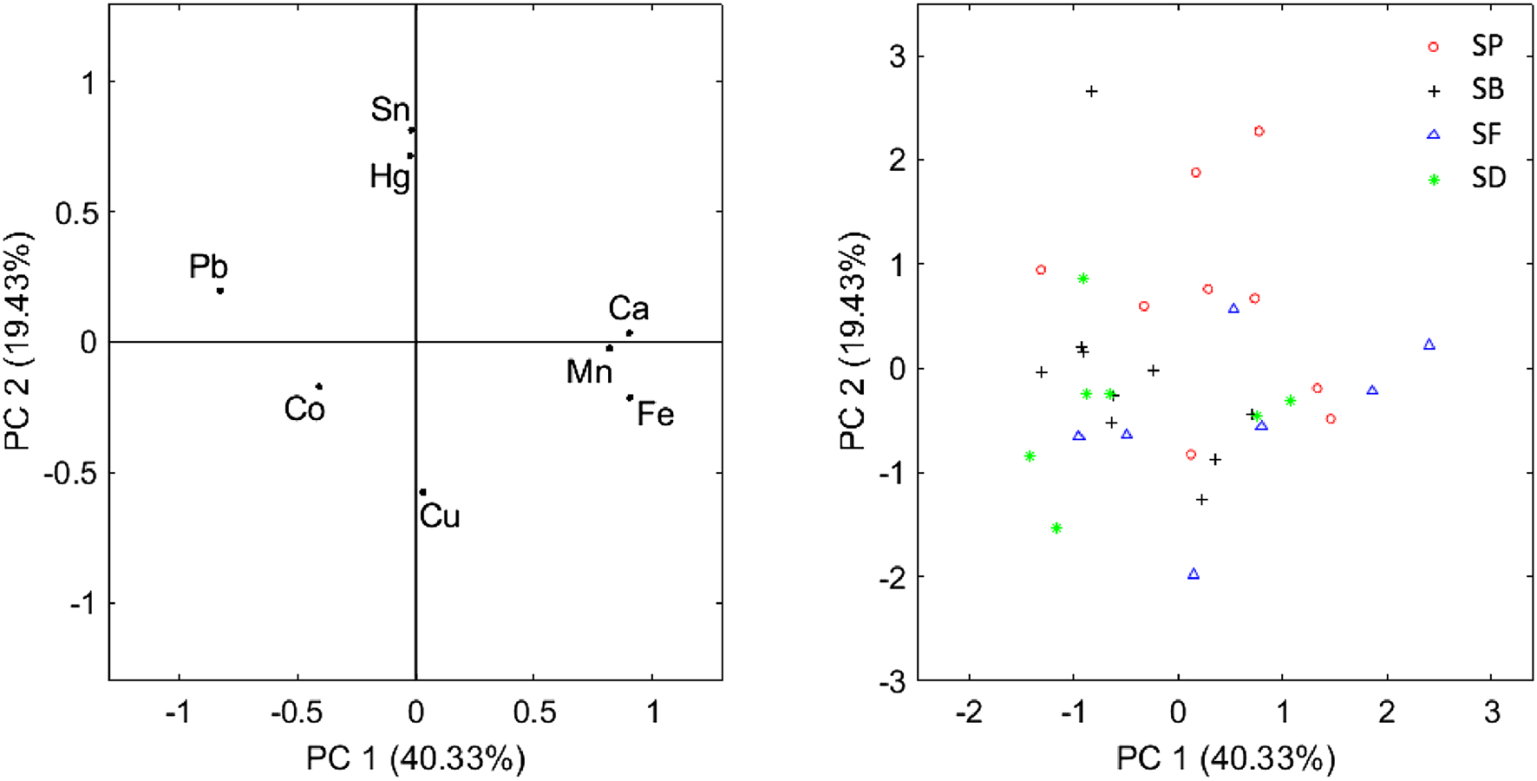
PCA scores (left) and results of the PCA analysis applied to the pigments of vegetation, rocks, and ground (right).

The green colour of the vegetation was achieved with a mixture of azurite, yellow earth and lead white, while red pigments, umber and earths were added for different tonalities. Additionally, smalt was also applied in the vegetation for the *SD* and *SB* paintings. The rocks and ground were mainly painted with earths and umber together with azurite, smalt and vermilion in different amounts depending on the tonality of brown colour achieved. However, vermilion was not found in these areas of the *SD* painting, but a red lake could have been used instead because some measures showed intense Ca peaks, while *SP* showed the absence of smalt. In addition, lead white, lead–tin yellow and bone black were added for the lights and shades, as has already been commented for other areas of the paintings. On the other hand, the inscriptions of the names of the saints on the rocks were made with earths, umber, a copper-based pigment, bone black, and vermilion, the last one added only in *SP* and *SB* with a very large difference in quantity. Figure 8 shows the PCA of the chemical elements. Due to the wide variety of colours and tonalities, it is difficult to group the points, but some important conclusions can be reached. Most of the *SP* points are in the upper part of the distribution, indicating a greater use of vermilion and lead–tin yellow with respect to the other paintings, where the use of lead white and smalt predominate.

#### Interventions

XRF was also used to identify pigments in areas where UV and IRR revealed later interventions. Most of these areas had a low concentration of Zn and Ti, which verified the existence of retouches with modern pigments: zinc white (ZnO) and titanium white (TiO_2_). Zinc white was commercialised in the second half of the nineteenth century to replace the poisonous lead white, while the titanium white appeared around 1920^20^. Ti and Zn were generally identified in the same analysed points; therefore, they might belong to an intervention from the twentieth century, or the same areas of the paintings were showing damages and had to be restored in different times. On the other hand, the *SD* painting showed the presence of Cr in two analysed points, which is identified with a nineteenth century chrome green pigment. We also found interventions prior to the nineteenth century, discovering several plaster-based fillings (clear intervention) polychromed with traditional pigments. Finally, the bluish wings of the angel showed a very high amount of Fe, which may indicate the use of earths or even the Prussian blue pigment. The Prussian blue was already known from the eighteenth century, so it could belong to any of the interventions mentioned above.

## Discussion

The analysis of the materials showed important differences in the use of pigments between the paintings. The presence of vermilion in the skin tones was much higher in *SD* and *SP*, presenting a more reddish colour of the skin. On the other hand, the shades of the tunics and the red tonalities of the sky were made with different dark and red pigments, respectively, in each painting. In addition, the landscape was painted with a greater use of vermilion and lead–tin yellow in *SP* painting, while for the other three paintings there is a larger presence of smalt than of azurite. All these differences could indicate different authors, as suggested by art historians who already indicated that the paintings would have been performed by the workshop.

Of the four canvased studied, *SP* stands out, revealing several distinctive characteristics that could indicate that it was executed by Zurbarán’s hand. First, in this painting, the preparation layer was the finest among all four canvases, revealing a skilled artist. Second, the high presence of azurite also called the attention. Being an expensive pigment, obtained from a semi-precious mineral, it was used not only in several colour layers in a higher amount than in the other three paintings, but also in the preparation layer. Adding a small amount of azurite to a mixture of other pigments as in greens, observed in other three paintings but also mixed with smalt, does not suppose a large cost, but covering the entire areas with azurite as the sky would have represented a larger expense. Therefore, it is natural to think that the greater use of an expensive pigment would have been carried out by Zurbarán himself rather than by his workshop. Another particularity is the presence of minium, not found in the other three canvases, corresponding to the palette of some other Zurbarán artworks^27–31^. Furthermore, the colour intensity and vivacity of *SP* is very different from other three paintings, as revealed by the use of vivid red (vermilion), yellow (led-tin yellow) and blue (azurite) colours, although in small amount. No doubt, this canvas shows the best visual result and corresponds to what is known about Zurbarán: the preparation and priming characteristic for the Spanish Golden Age; a convincing drawing with almost no modifications; the painting technique used, combining basic colour layers with final, very transparent glazes; his careful brushstroke; the characteristic tonalities of the colour palette that correspond to his later works; the precise face modelling with fine colour transitions and the soft texture of the draperies. All this reveals a highly skilled artist and strongly points toward the hand of Zurbarán. Also stylistically, this painting is very close to other works signed by him, where the same models were used in different positions. Altogether, *SP* presents a finer brushstroke, a better technique and a higher knowledge on pigments with difference to the other three saints, confirmed by this study.

## Conclusions

The study of the material of four paintings attributed to the Zurbarán Workshop from the collection of the Archbishop’s Palace showed some variations in the use of pigments to achieve similar colours and tonalities in some areas. The preparatory layer was made with gypsum, calcite, earths, and lead white, but azurite and bone black were also added in some areas of the paintings of *SP* and *SF*, respectively. A lead white primer was applied locally, while the pigments used in the four paintings correspond to those commonly used during the seventeenth century: lead white (Pb), yellow and red earths (Fe), lead–tin yellow (Pb, Sn), vermilion (Hg), red lake (Ca), minimum red (Pb), smalt (Co, K, Ni, As, Bi), azurite (Cu), umber (Mn, Fe) and bone black (Ca). Furthermore, the green colour was achieved with a mixture of blue and yellow pigments, as characteristic for other contemporary Spanish artists.

*SP* showed distinctive characteristics that were not found in the other three paintings. The more extensive use of azurite in the preparation and in several colour layers resulted, among others, in the preservation of the blue colour of the sky, which did not suffer the alteration of the smalt that happened with the other paintings. However, the UV images presented a blue lighting in the sky produced by the smalt, which would offer an original vision of the blue colour of these areas before darkening. In addition, the greatest use of vermilion and lead–tin yellow for both reddish skin tones and highlight effects showed the best visual results, while minium red was only found in this painting. All these differences support the initial hypothesis made in private studies of the Archbishop’s Palace that the *Saint Peter Martyr* painting was executed by Zurbarán, while the other three paintings were made by his workshop. These characteristic features regarding the use of pigments will help to identify more paintings by Zurbarán and compare his techniques and those of his workshop with other contemporary artists.

## Supplementary Information

Supplementary Information.

## Data Availability

All data generated or analysed during this study are included in this published article and its supplementary information files.
