# Supplementary figures and images for: Zurbarán attribution hypothesis supported by pigment analysis and multiband images observation of four paintings by his workshop
Source: Sci Rep. 2023 Jan 16;13:844. doi: 10.1038/s41598-023-27677-2 (PMC9842728; doi:10.1038/s41598-023-27677-2)

**ZURBARÁN: San Bruno**

**PUNTOS**

**
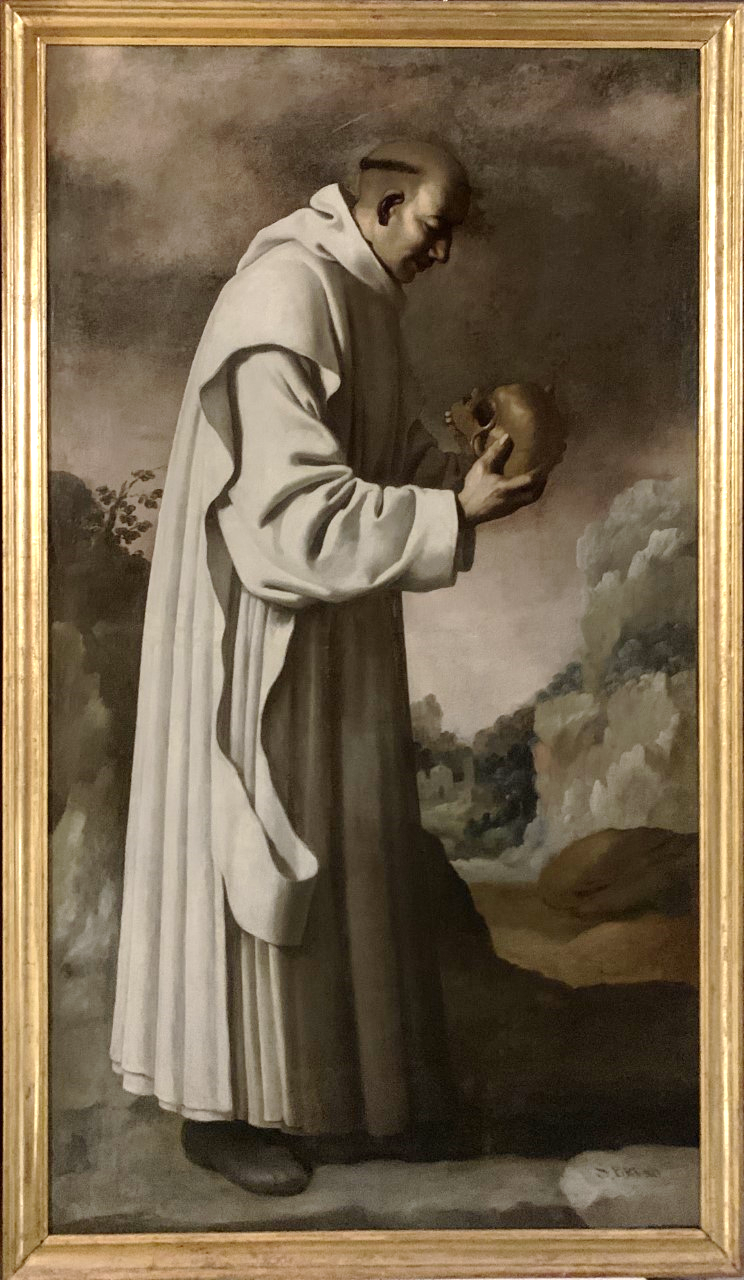
**

**26**

**19**

**25**

**24**

**23**

**22**

**21**

**20**

**18**

**17**

**16**

**15**

**14**

**13**

**12**

**11**

**10**

**9**

**8**

**7**

**6**

**5**

**4**

**3**

**2**

**1**

Supplement: Supplementary file 1 — Supplementary Information. [file 41598_2023_27677_MOESM1_ESM.zip › Raw_Data/San Bruno/San Bruno PUNTOS.docx]

**ZURBARÁN: San Francisco de Asís**

**PUNTOS**

**
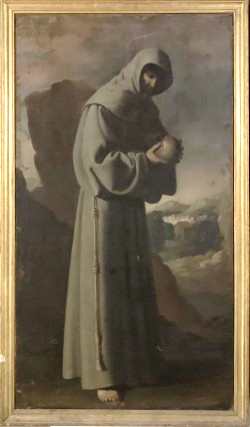
**

**26**

**25**

**24**

**23**

**22**

**21**

**20**

**19**

**18**

**17**

**16**

**15**

**14**

**13**

**12**

**11**

**10**

**9**

**8**

**7**

**6**

**5**

**4**

**3**

**2**

**1**

Supplement: Supplementary file 1 — Supplementary Information. [file 41598_2023_27677_MOESM1_ESM.zip › Raw_Data/San Francisco/San Francisco de Asis PUNTOS.docx]

**ZURBARÁN: San Pedro Mártir**

**PUNTOS**

**
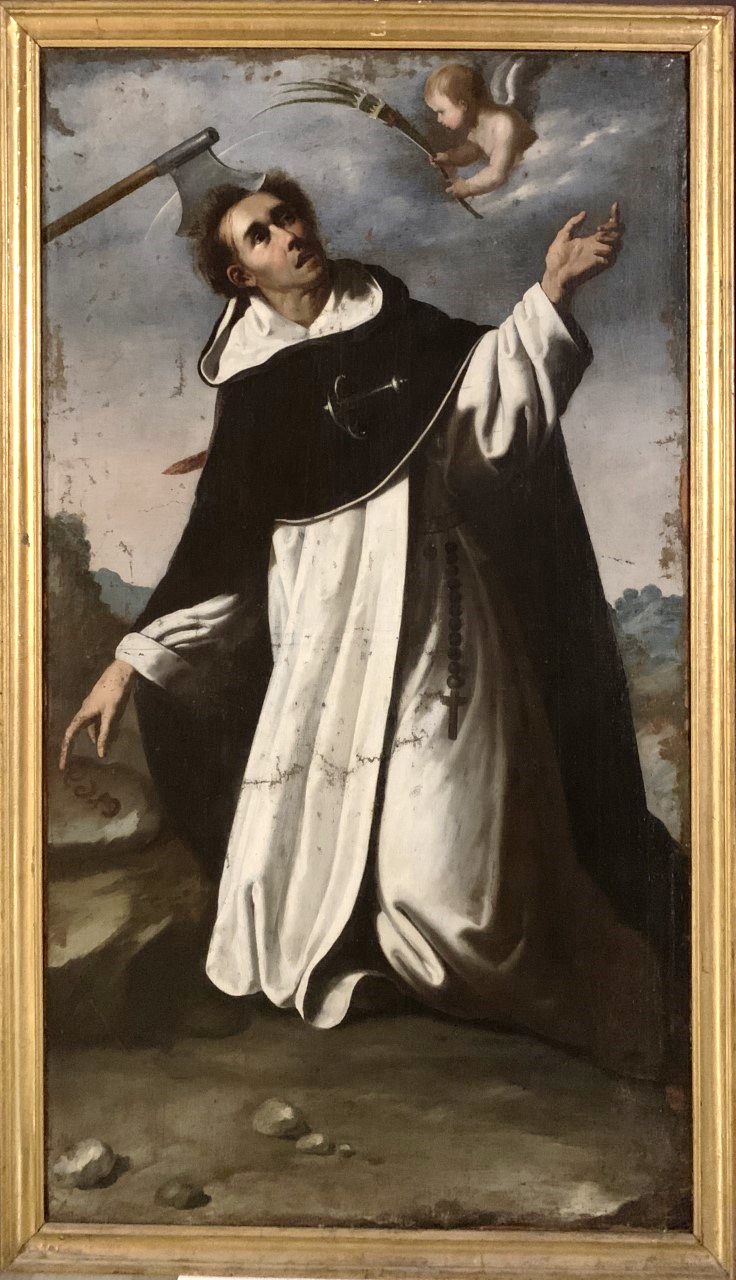
**

**43**

**42**

**41**

**40**

**39**

**38**

**37**

**36**

**35**

**34**

**33**

**32**

**31**

**30**

**29**

**28**

**27**

**26**

**25**

**24**

**23**

**22**

**21**

**20**

**19**

**18**

**17**

**16**

**15**

**14**

**10**

**13**

**12**

**11**

**9**

**8**

**7**

**6**

**4**

**5**

**3**

**2**

**1**

Supplement: Supplementary file 1 — Supplementary Information. [file 41598_2023_27677_MOESM1_ESM.zip › Raw_Data/San Pedro/San Pedro Martir.docx]

**ZURBARÁN: Santo Domingo**

**PUNTOS**

**
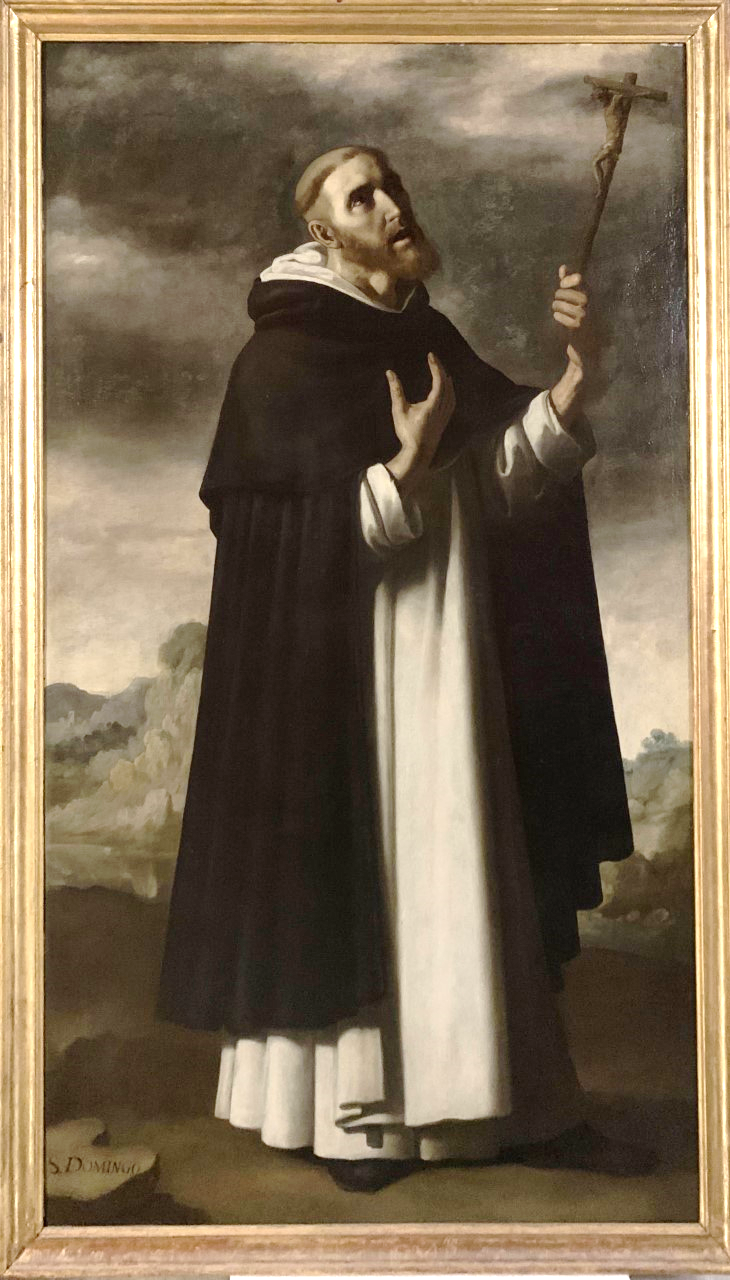
**

**9**

**7**

**24**

**23**

**22**

**21**

**20**

**19**

**18**

**17**

**16**

**15**

**14**

**13**

**12**

**11**

**10**

**8**

**6**

**5**

**4**

**3**

**2**

**1**

Supplement: Supplementary file 1 — Supplementary Information. [file 41598_2023_27677_MOESM1_ESM.zip › Raw_Data/Santo Domingo/Santo Domingo PUNTOS.docx]
